# Conformational space and vibrational spectra of 2-[(2,4-dimethoxyphenyl)amino]-1,3-thiazolidin-4-one

**DOI:** 10.1007/s00894-014-2366-6

**Published:** 2014-07-15

**Authors:** Alicja Nowaczyk, Marcin Kowiel, Andrzej Gzella, Łukasz Fijałkowski, Volodymyr Horishny, Roman Lesyk

**Affiliations:** 1 Department of Organic Chemistry, Faculty of Pharmacy, Collegium Medicum in Bydgoszcz, Nicolaus Copernicus University, Dr. A. Jurasza 2, 85-094 Bydgoszcz, Poland; 2 Department of Organic Chemistry, Poznan University of Medical Sciences, ul. Grunwaldzka 6, 60-780 Poznań, Poland; 3 Department of Pharmaceutical, Organic and Bioorganic Chemistry, Faculty of Pharmacy, Danylo Halytsky Lviv National Medical University, Pekarska 69, 79010 Lviv-10, Ukraine

**Keywords:** ab initio calculations, FTIR spectral characteristics, Tautomerism, 1,3-thiazolidin-4-one, X-ray analysis

## Abstract

In this work we present the results of a study of the X-ray structure of 2-[(2,4-dimethoxyphenyl)amino]-1,3-thiazolidin-4-one. Using the FTIR spectra in solid state and results of ab initio calculations we explain the issue of the tautomerism of this molecule. The compound is shown to exist as the *2-amino* tautomer rather *2-imino* tautomer. Here we consider eight possible tautomers. On the basis of the vibrational spectra we can eliminate five possible tautomers, as not existing in the solid state. As the most possible tautomeric form we have found *keto 2-amino* form.

**Electronic supplementary material:**

The online version of this article (doi:10.1007/s00894-014-2366-6) contains supplementary material, which is available to authorized users.

## Introduction

The 1,3-thiazolidin-4-one as saturated form of thiazole ring is heterocyclic nucleus that has sulfur and nitrogen atoms at position 1 and 3, and a carbonyl group at position 4 respectively. It represents an important structural moiety included in many compounds of pharmacological importance and have been subjected to extensive study in recent years [1]. The thiazolidinone ring has been incorporated into a broad range of known biologically active compounds, either as a substituent group or a replacement of another ring. These have motivated researchers to synthesize compounds containing the mentioned heterocyclic moiety. The thiazolidin-4-one scaffold is very flexible despite the presence of sulfur, nitrogen, and oxygen atoms. Combination of such molecular flexibility and fundamental heteroatoms yield almost all types of biological activities. Consequently the thiazolidin-4-one fragment can be found in a number of clinically used drugs. Biological screening has demonstrated activities such as bactericidal, pesticidal, fungicidal, insecticidal, antiviral (anti-HIV), antidiabetic, anticonvulsant, tuberculostatic, antiinflammatory, antithyroidal, anticancer, antithyroidal, and immunostimulant [2–6]. According to this the thiazolidin-4-one fragment is considered to be a wonder nucleus. Some thiazolidin-4-ones have potentiation of pentobarbital-induced sleeping time, antihypertensive [3, 5, 4]. It is worth mentioning that substituents in the 2-, 3-, and 5-positions may be varied, but the greatest difference in structure and properties is exerted by the group attached to the C2-position [7–10]. Thus a few derivatives with C2 and N3 substituted positions and the presences of electron-withdrawing substitution on aromatic ring on C2 position of thiazolin-4-one presenting varied degrees of inhibition against Gram-positive and Gram-negative bacteria showing inhibition as good as the standard drugs used. On the other hand, retrospective analysis of these compounds showed that anticancer activity increases while replacement from cycloalkyl moiety to heteryl moiety in position C2 [2].

**Fig. 1:**
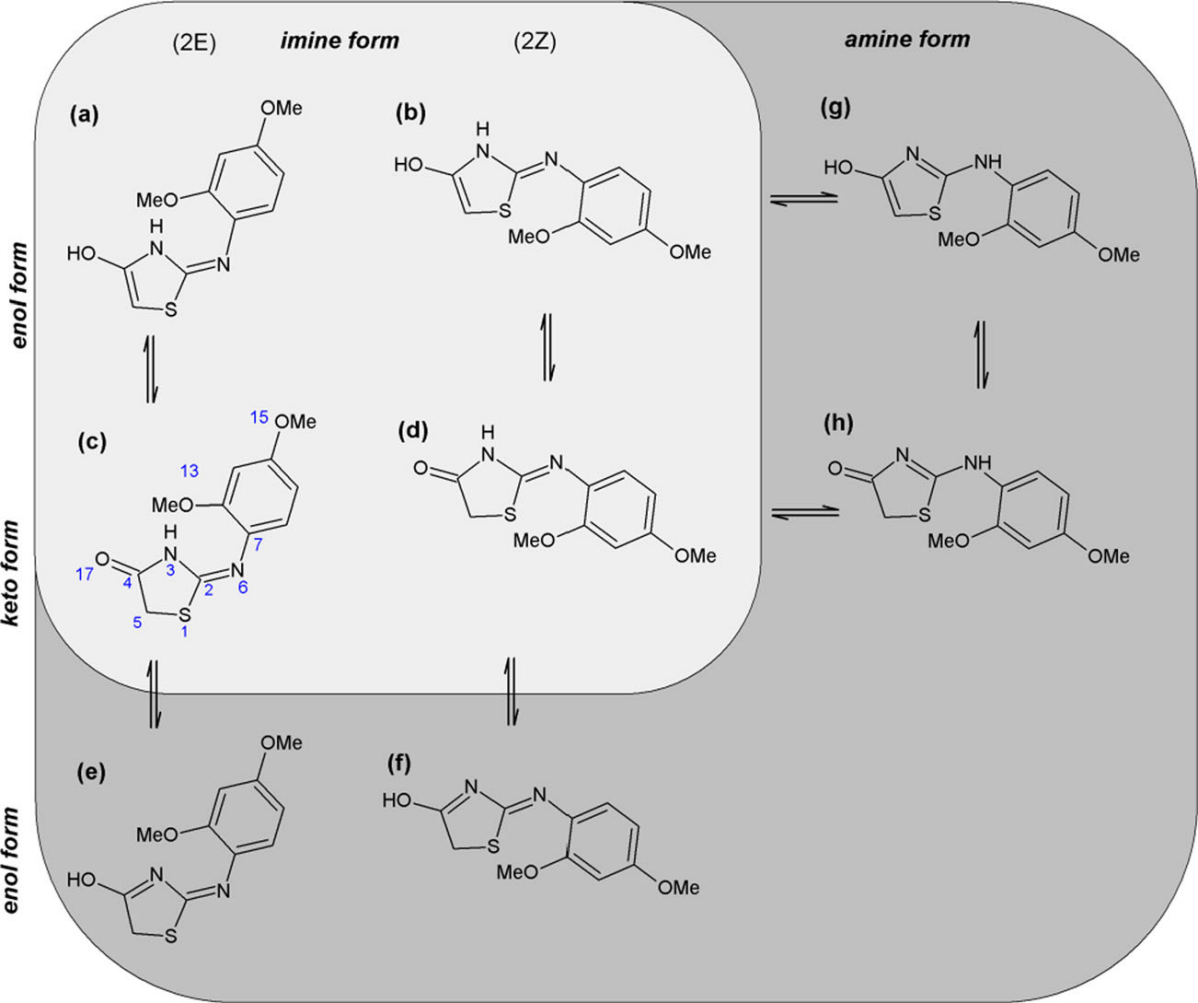
The 2-[(2,4-dimethoxyphenyl)amino]-1,3-thiazolidin-4-one for the *imino* ⇋ *amino* and *keto* ⇋ *enol* tautomeric forms

A detailed study of the characteristic bands in the infrared spectra of several 2-substituted thiazolidin-4-ones has been done in the past [11–14, 10]. The *imino-amino* tautomerism of 2-substituted thiazolidin-4-ones was studied by infrared spectroscopy [15, 16]. Taylor et al. [17] described the criteria for determining the *cis* and *trans* configurations of these compounds. The *cis* isomer is favored when H-bonding exist in the system otherwise the isomer is impossible. In other conditions, the *trans* isomer is the stable form. It was shown by the methods of IR (and NMR) spectroscopy that 2-aminothiazolin-4-one (“pseudothiohydantoin”) exists in an *imine* form in the crystalline state and in solutions in dimethyl sulfoxide, water, and trifluoroacetic acid, and in this form the N3-C2 bonds are partially double [4]. Theoretical ab initio studies could supplement these measurements. Additionally, calculations of energy, atomic charges, minimum energy structures, geometry, and natural bond orbital (NBO) could indicate the electronic density distribution of each atom. Nowadays, the formulation of predictive theoretical models is necessary in pharmaceutical research because of the considerable reduction of cost and avoidance of animal testing. The constitution of the 2-iminothiazolidin-4-one, also known as pseudothiohydantoin [18], was first proposed by Liebermann et al. in 1879 [19, 20]. However, the discussion on the tautomerism of this molecule continued for more than a century [14]. Their tautomerism was studied experimentally by different authors. From the information available in the literature the 2-iminothiazolin-4-one and its 2-aryl derivatives are shown to exist as the *2-amino* tautomer rather than *2-imino* tautomer [2, 21, 22]. The title compound can exist as eight possible tautomers, Fig. 1 a-h. In the solid state its infrared spectrum shows the presence of a carbonyl absorption and the absence of an hydroxyl absorption, thereby eliminating five possible tautomers (a, b, e-g). In this work we present theoretical and experimental investigation on the X-ray crystallography and IR spectra of the above mentioned structures with ab initio methods.

## Materials and Methods

### Synthesis procedure

The compound described in this paper was synthesized by the reaction protocols typically used for obtaining 2-arylamino-1,3-thiazolidin-4-one derivatives [23]. The starting 2-carbethoxymethylthio-2-thiazolidin-4-one was obtained by the reaction of 2-thioxothiazolidin-4-one triethylammonium salt with ethyl chloroacetate in acetone. Reaction of 2-carbethoxymethylthio-2-thiazolidin-4-one with a 2,4-dimethoxyaniline in refluxing ethanol provided target 2-[(2,4-dimethoxyphenyl)amino]-1,3-thiazolidin-4-one with 80 % yield. The product formed was filtered, washed, dried, and crystallized from n-butanol.

### Structural analysis

The structure of the studied compound was confirmed using the X-ray crystallography and IR spectroscopy. The X-ray diffraction measurements were carried out using Agilent Xcalibur A diffractometer, detailed crystallographic information is included in Supporting information. The mid infrared region the FT-IR spectrum of 2-[(2,4-dimethoxyphenyl)amino]-1,3-thiazolidin-4-one was recorded in KBr pellet. The spectrum was taken with a Bruker IFS 66v/S FT-IR spectrophotometer equipped with a DTGS detector; resolution 2 cm^−1^. The Happ–Genzel apodization function was used [24].

#### Computations

The title compound can exist in several possible tautomers shown in Fig. 1 a-h. In this work the 3-dimensional structures of the 1,3-thiazolidin-4-one tautomers in their neutral state were obtained by the DFT [25] approach utilizing Becke’s three parameter functional [26] with the Vosko et al. [27] local and Lee et al. [28] non-local correlation, abbreviated as B3LYP. The ab-initio quantum chemical calculations using standard Pople’s 6-31G(d,p) basis set [29] including d polarization functions for carbon, nitrogen, and oxygen and p polarization functions for hydrogen atoms. The rotations about the C2-N6 (Fig. 1 g-h.), N6-C7 (Fig. 1 a-f.) bonds respectively were taken into account. We have calculated the rotational energy barrier by steps of 10° around. We take the X-ray structure of the 2-[(2,4-dimethoxyphenyl)amino]-1,3-thiazolidin-4-one (Fig. 1h.), as starting geometries for all studied compounds. All eight tautomers presented in Fig. 1 were considered in our study. All the molecules were geometry-optimized until the root-mean-square (RMS) gradient value was smaller than 10^−6^ a.u. To structurally characterize the molecule in detail, a systematic investigation of its potential energy surface was undertaken at the DFT(B3LYP)/6-31++G(d,p) level of approximation [30]. Later, using the surface data generated from Gaussian checkpoint files, and GaussView 4.1 software, the distribution of charge in a molecule was calculated. To obtain a 3D plot of the MEP, the electrostatic potential cube file was calculated from total SCF density. The contour maps of the electrostatic potential were then drawn using a distance between grid points of 0.02 Å and the isovalues 0.0004. The Gaussian software suite was used to calculate the electrostatic potential maps and surfaces as the distribution of the potential energy of a unit positive charge in a given molecular space, with a resolution controlled by the grid density.

The vibrational wavenumbers were calculated at the DFT(B3LYP)/6-31++G(d,p) level of approximation. It is well known in the quantum chemical literature that among the available functionals, the B3LYP functional yields a good description of harmonic vibrational wavenumbers for small- and medium-sized molecules.

All calculations were performed using the Gaussian 03 program [32]. The visualizations were prepared by use of the Gaus-View 4.1 [33]. Theoretical calculations were conducted on the Cluster Supercomputer at Nicolaus Copernicus University Computational Center.

## Results and discussion

### Tautomeric forms

The tautomeric forms of the studied compound together with numbering system are shown in Fig. 1. The 2-substituted 1,3-thiazolidin-4-one can exist as *2-amines*, in which the electron pair of the sp^2^-hybridized nitrogen atom is in conjugation with the four π electrons of the C = C and C = N double bond (Fig. 1g). There also exist the *2-amine* tautomer in which the electron pair of the sp^2^-hybridized N atom is conjugated with four π electrons of the C = O and C = N double bonds respectively (Fig. 1h). Additionally there are two tautomers of *2-imine* in which there is conjugation including π electrons of the two C = N double bond (Fig. 1 e-f). There are as well four tautomers having no conjugated double bonds whatsoever (Fig. 1 a–d). On account of the mobility of the hydrogen atom of the CH_2_ group in position 5 the pair of a-c, b-d, g-h can be in tautomeric equilibrium. Simultaneously on account of the mobility of the hydrogen atom of the NH group a (or b) -g, and c (or d) -h and c-e, d-f can be in tautomeric equilibrium. Theoretically, E/Z isomerism is possible across the double bond a-b, c-d, e-f and the molecule may exist in the *2-imino* form with the possibility of interconvertion via *2-amino tautomer* (e-h) as depicted in Fig. 1. According to Taylor and coworkers and Steel et al. [31, 11, 12, 17] the *Z*-isomer predominates when H-bonding occurs, otherwise this isomer is impossible. In other conditions, the *E* isomer is the stable form.

### 2-*amino*(*imino*)-1,3-thiazolidin-4-ones -CSD studies

In our laboratory, the crystal structures of various thiazolidin-4-ones have been investigated in the past few years [10, 23, 7, 32, 8, 33, 34]. A lot of 2-*amino*(*imino*)-1,3-thiazolidin-4-ones have been found in the *Crystal Structure Database* (CSD version 5.35, Jun 2014) [35]. The CSD searches for 2-*amino*(*imino*)-1,3-thiazolidin-4-one derivatives with or without C-5 and N-3 substituted positions were done for the neutral forms of the species. The list of all found structures and geometrical specifications can be found in Tables S1-S7 in Supporting information. The search has resulted in 97 hits, of which 30 hits having molecules containing N3 or N6 secondary amine group (Table S1-S2). In the case of 21 possessing substituent at the C-2 and C-5 positions of the thiazolidin-4-one moiety (16 bearing 2-*amino* Table S1 and five 2-*imino* forms Table S2 respectively). In the case of the remaining 67 hits, the structures contain tertiary amine group (Table S3-S4) of which 49 hits having substituent at the 3-position at the heterocyclic ring (Table S3) and 18 having substituent at the N6 position (Table S4). Thus the search showed that the population of the structure containing tertiary amine group and substituents at the C-2 and C-5 position of the thiazolidin-4-one results in 37 hits. It can be remarked that 22 hits possessed substituents at the C-2 and C-5 position and amine N3 atom and 15 hits which has a substituent at the C-2 and C-5 position and amine N6 atom. In the literature attention was mainly drawn to the *imine ⇋ amine* conversion. However, the majority of tautomeric equilibrium of heteroatomic molecule are prototropic, i.e., involve proton migration between (i) carbon and O center; (ii) nitrogen and O, N centers. From a chemical point of view, another tautomerism such as *keto ⇋ enol* is also possible (Fig. 1.). Among 2-*amino* (*imino*)-1,3-thiazolidin-4-ones deposited in the CSD database, though, none adopts the *enol* tautomeric form. Based on 20 derived 2-*amine* structures deposited in the CSD database the average bond length values for C2–N3 estimated as 1.325(1)Ǻ and C2–N6 as 1.315(2) Å (Supporting information, Table S5) were calculated. Comparing these values with normal length of double bond for C = N (1.279(1) Å) and single bond Csp2–N (1.383(1) Å) [36, 37] (Supporting information, Table S6) indicates that the first is extended in comparison to the length of the double bonds by 0.046 Å, and the next is shorter by 0.068 Å with respect to the single bond length. These results show that these two bonds have a partial double-bond character in the molecules. Moreover the average length of C2–N3 and C2–N6 bonds were also calculated for eight derivatives of *2-imine*. The length of C2–N3 bonds was estimated as 1.374(3) Å and C2–N6 bond as 1.280(2) Å (Supporting information, Table S5). The former is similar to the typical single C*sp* ^2^−N bond length. The latter is close to C=N bond length. The mutual position of thiazolidin-4-one in *E*/*Z* point of view may be described by the value of S1–C2–N6–C7 torsional angle (Supporting information, Table S7). The separate analyses revealed that, in the majority of hits, the torsion angle values are around 0° and exhibit more or less coplanar conformation. Such a predominant conformation found in CSD database is 2*Z*.

### Crystallographic and calculated structures

**Fig. 2:**
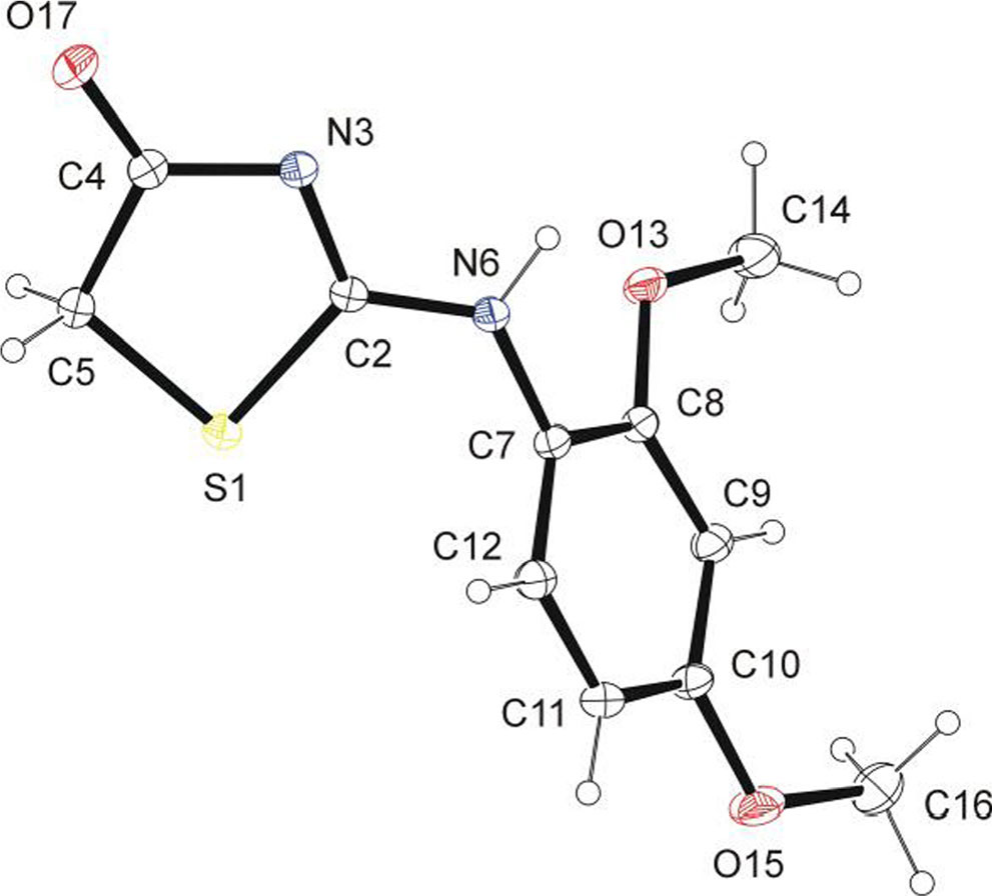
X-ray crystal structure (ORTEP plot) of 1. The crystallographic data in the CIF form are available as electronic supplementary information from the Cambridge Crystallographic Database Centre (CCDC 1003815)

**Fig. 3:**
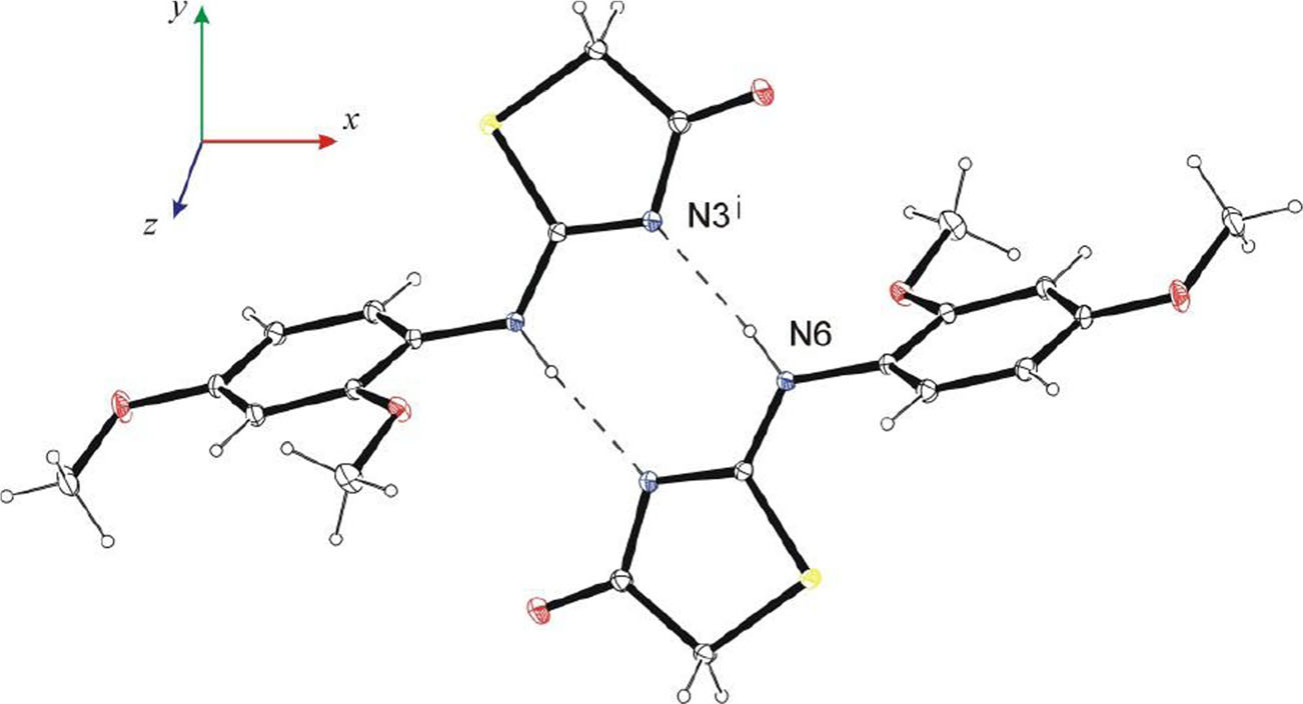
The centrosymmetric hydrogen-bonded dimer of 1 h [symmetry code: (**i**) 0.5-x,0.5-y,-z]

X-ray analysis revealed that compound 1 occurs in crystal in 2-*amine* form (Fig. 1h, 2 and Table 1). This was confirmed by the presence of hydrogen atom connected to the exocyclic nitrogen N6 and similar values of C2−N3 and C2−N6 bond lengths [1.3265(11) and 1.3247(11) Å, respectively], which is a typical observation for this tautomeric form. It can be remarked that both bonds mentioned above reveal partially double character. They are shortened by about 38 and 39σ with respect to the normal single C*sp* ^2^−N length 1.383(2) Å [37, 36]. On the other hand, they are lengthened by about 32 and 31σ in comparison with the literature double C=N bond length 1.279(1) Å [37, 36]. The bond C2−N6 displaying partially double character hinders the rotation of N6−C7 bond. As a result, C7 only slightly sticks out of the least-squares plane of thiazolidin-4-one. The observed torsional angle S1−C2−N6−C7, 2.43(12)º indicates that S1−C2 and N6−C7 bonds are in synperiplanar orientation. The observed conformation create favorable conditions to form N6−H6 · · · N3^i^ [(i) 1-x,1-y,-z] hydrogen bonds in crystal which connect the molecules related by the inversion center into dimers (Fig. 3). In the latter thiazolidinone moieties are arranged coplanar. This is different from the phenyl and thiazolidinone systems, which form a dihedral angle 59.05 (4)°. The reason for such a large dihedral angle should be seen in the presence of the methoxy group in the *ortho* position of the phenyl ring, preventing flattening of the molecule.

**Fig. 4:**
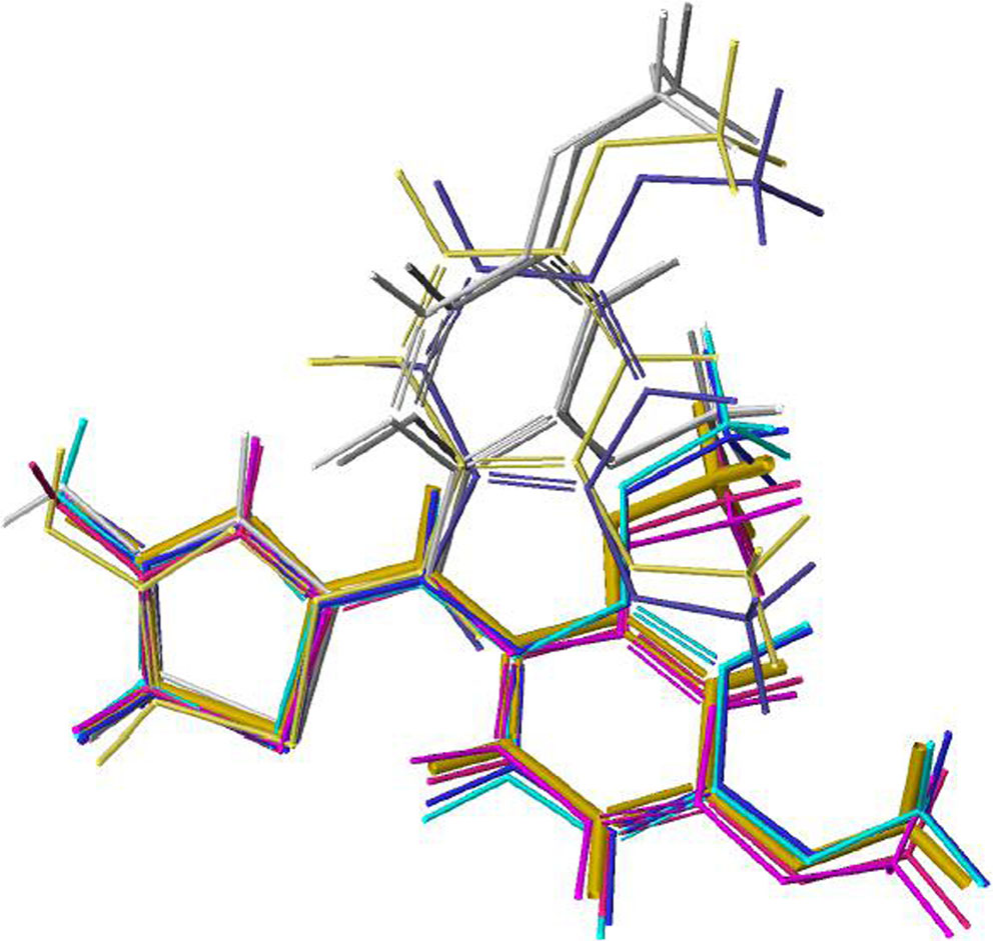
Frontal view of the most stable calculated structures obtained by superimposing with the X-ray structure overlapped by calculated structures

The optimized structure produced is very similar to the experimental one. Both the optimized and experimental structures of the title molecules were compared by superimposing them using a least-squares algorithm that minimizes the distances between the corresponding non-hydrogen atoms as shown in Fig. 4. The color code of these compounds are: yellow for the crystallographic structure, and the other colors represent calculated structures such as: platinum a, red b, violet c, purple d, gray e, blue f, green g, lemon h.

### Potential energy studies

Rotation around the C2–N6 and C7–N6 bonds is possible exclusively in *amine* and *imine* respectively. The analysis of the adequate potential energy surfaces by means of redundant coordinate technique indicate the existence of eight absolute minima, which correspond to the most stable conformers of investigated structures. The matrix of energy differences between every pair of structures investigated in this paper is given in Table 2. Taking into account the calculated relative energy barriers for the different conformers the most stable in the gas phase are conformer h and d, due to the highest energy differences of other compounds (Table 2). However the minimum differences of energy was observed in d-h pair, i.e., about 2 kcal mol^−1^ which suggests that 2Z *imine* and *amine* counterpart may not be experimentally accessible as isolate species. Nowadays the most information on conformational isomerism comes from single-crystal X-ray diffraction studies. According to the experimental observations the separation of the conformers as chemical isolate form can be accessible if ΔE ≈ 20–30 kcal mol^−1^. As it can be see in Table 2 the *keto* tautomers are generally lower in energy than their *enol* counterpart by less than 20 kcal mol^−1^. Structural analysis of the α-substituted *imines* and *amines* in terms of the functional group suggests that the C=NH and C= C-NH groups correspond to *keto-enol* tautomerization of C=O and C= C-OH groups [38]. Since the *keto* tautomers are generally lower in energy than their *enol* counterpart, it is reasonable to predict that the *amines*, in general, will be more stable than the *imines*. This fact is reflected in the CSD database. Moreover our calculations show that the most stable conformation is *amine* form h, which is in agreement with the crystallographic study. Tables 1 and 2

**Table 1:** Crystal data and structure refinement for 2-[(2,4-dimethoxyphenyl)amino]-1,3-thiazolidin-4-one (1 h)

Formula	C_11_H_12_N_2_O_3_S
Formula weight	252.29
Temperature/K	130(2)
Wavelength/Å	0.71073
Crystal system	Monoclinic
Space group	*P*2_1_/*c*
*a*/Å	14.11229(15)
*b*/Å	10.02574(14)
*c*/Å	8.12131(12)
*α*/º	90.00
*β*/º	96.8434(11)
*γ*/º	90.00
V/Å^3^	1140.87(3)
*Z (Z’)*	4 (1)
*D*_*c*_/g cm^−3^	1.469
*μ*/m m^−1^	0.281
*F*(000)	528
Crystal size/mm	0.60*0.25*0.20
*θ* range	2.50–32.53º
Max/min. indices *h*, *k*, *l*	−20 ≤ *h* ≤ 21,-14 ≤ *k* ≤ 9, −12 ≤ *l* ≤ 9
No. of data collected	11162
Independent reflections	3832 (*R*_int_ = 0.0181)
Completeness to *θ*_max_ = 25.00°/%	100
Restraints/parameters	0/161
Goodness-of-fit on *F* ^*2*^	1.030
Final *R* indices [*I* > 2*σ*(*I*)]	R1 = 0.0298, wR2 = 0.0797
R indices (all data)	R1 = 0.0340, wR2 = 0.0826
Largest diff. peak and hole/eÅ^3^	0.476 and −0.268

**Table 2:** The energy barrier between structures investigated in this paper ΔE [kcal mol^−1^]

	a	b	c	d	e	f	g	h
a	0	−1	−12	20	12	−5	10	22
b		0	−11	21	13	−5	11	23
c			0	32	24	6	22	34
d				0	−8	−26	−10	2
e					0	−18	−2	10
f						0	15	28
g							0	13
h								0

### Tautomers and biological activity

**Fig. 5:**
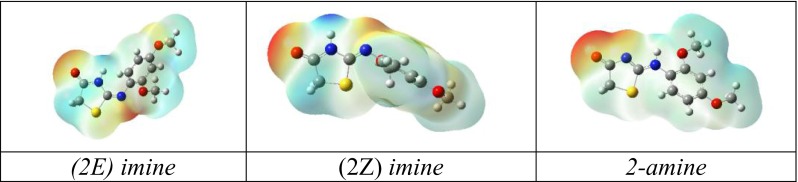
The view of calculated contour electrostatic potential maps for typical tautomers presented in crystal state (c, d, h respectively)

Taking into account the molecular interactions, it would be more informative to discuss some biologically important facts. The heteroatomic systems with 4-, 5-, 6-membered rings are common structures in many drugs. Most of them occur in two or more tautomeric structures. The prevalent form of tautomerism is prototropy which refers to the relocation of a proton. Various tautomers of the same compound could differ in biological activity, also it is important to identify potential for tautomerization in heteroatomic systems. A drug with possible amine to imine (NH to N) tautomerism is a change that decreases the aromaticity of the heteroatomic system. Exemplary drugs could reveal probable NH to O tautomerism, commonly existing as the amide tautomers. Therefore, the centers for the formation intermolecular hydrogen bonds are provided. Many antiviral agents along with drugs used in treatment of HIV infections exhibit the potential NH to N and NH to O tautomerism [39]. The structure activity relationship revealed that thiazolidine ring is essential for antibacterial and antiviral activity [40]. From this perspective, studies of the literature data revealed that the negative charges of the oxygen of C=O group and positive charge of nitrogen in NH contribute positively in favor of an antibacterial activity. It was hypothesized that difference in charges between two heteroatoms of the same dipolar pharmacophore site (X^δ-^–Y^δ+^) may facilitate the inhibition of bacteria, more than viruses growth. Additionally this is in good agreement with the mode of antibacterial action of the compounds bearing (X^δ-^–Y^δ+^) pharmacophore site [41]. The antiviral activity is related to possible secondary electronic interaction with the positively charged side chains of the virus target(s). It was further found that the activity increases with increase in negative charge of one heteroatom of the common pharmacophore fragment of the potential tautomer. This means, that topologically related pairs of atoms need to be close to each other to promote biological activity. The heterocyclic ring in adjacent position of NH could generate two *imino*-*amino* tautomeric forms, and two distinct four-membered pharmacophore sites are conducive to the activity of both antibacterial (O ^δ−^–NH^δ+^) and antiviral activity (O ^δ−^–N^δ−^). A common way of visualizing the distribution of charge in a molecule is to map the electrostatic potential in the form of a 3D plot (or a 2D contour plot) of the electrostatic potential distribution (MEP). Regions of the electron density surface that are more negative than others in an MEP are colored red. Regions in the MEP that are less negative (or positive) are blue. The color spectrum indicates the trend in charge from most negative (red) through green and yellow (neutral) to positive (blue). It provides a visual method to understand the relative polarity of a molecule and serves as a useful quantity to explain hydrogen bonding, reactivity, and structure-activity relationship of molecules including biomolecules and drugs. It is the potential energy of a proton at a particular location near a molecule. In Fig. 5 the distribution of charge for a typical tautomer obtained in crystal state, i.e., c, d, h are depicted. The color code of these maps is in the range between −0.0697 a.u. (deepest red) and 0.0697 a.u. The analysis of conformational differences due to heteroatom interactions in tautomers a–h revealed a favorable (C = O–NH, C–OH–N) interaction in tautomer a-g, where tautomer h showed a repulsive (C=O–N) interaction. The *imine* tautomer can exist as E/Z stereoisomers as previously mentioned. As can be seen from the MEP, the compounds having E/Z configuration of *imine* has predicted higher probability of occurrence of antibacterial than its *amine* counterpart. In contrary, the *amine* spices have the higher chance to confirm antiviral activity in real pharmacological tests. The atomic charges for typical tautomers presented in crystal state (c, d, h respectively) are included in Table S8 in Supporting information.

### IR spectra

**Fig. 6:**
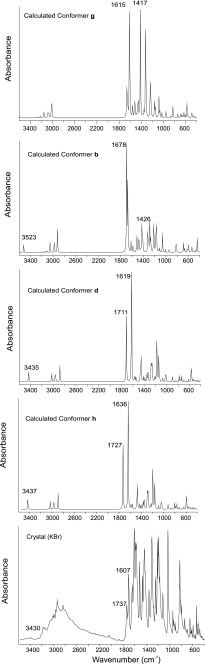
From bottom to top: calculated infrared spectra of conformers g, b, d, h. IR spectrum in the room temperature crystalline phase, as a KBr pellet

The experimental geometry was in good agreement with the here calculated ab initio results. Consequently it seems reasonable to use these geometries to calculate force constancies and theoretical IR spectra. The title compound has 29 atoms and hence gives 81 (3 N-6) fundamental modes of vibration, all of them are IR active. Since the vibrational wavenumbers calculated by DFT methods are higher than their precise values, they were scaled down. In order to correct the effects of basis set limitations (neglecting part of electron correlation) and anharmonicity effects several methods of correction were implemented. In this work the wavenumbers are scaled applying the wavenumber linear scaling procedure (WLS) [ν_obs_/ν_cal_ = (1.0087 – 0.0000163 × ν_cal_) cm^−1^] by Yoshida et al. [42]. The computed harmonic frequencies are scaled down by a factor, 0.985, obtained from linear fit of the calculated to experimental wavenumbers. The nature of stationary points on the potential energy surface was checked through the analysis of the corresponding Hessian matrix. The simulated spectrum reproduces very well the experimental spectrum, providing strong evidence for the presence of only *keto* forms in the solid state (Fig. 6). The comparison of experimental and calculated specific frequencies of important groups are collected in Table 3.

**Table 3:** Vibrational frequencies (cm^−1^) of the selected groups obtained from solid state spectra and calculated using ab initio method

Assignment	FTIR frequency	h	d	b	g
C = O	1737	1727	1711	–	–
C = N	1607	1636	1619	1678	1615
N–H	3430	3437	3435	3523	–
C–OH	–	–	–	1426	1417

X-ray crystallography

X-ray diffraction measurements were carried out on an Agilent Xcalibur A diffractometer [1]. The structure of 1 was solved by direct methods using the SHELXS-97 program [2]. Except for the amine H atom, which was refined freely the remaining H atoms were positioned geometrically and were refined within the riding model approximation, with C–H = 0.96 Å (CH_3_), 0.97 Å (CH_2_), 0.93 Å (C_*ar*_H), and *U*_iso_ (H) values were constrained to be 1.2 (1.5 for methyl group) times *U*_eq_ of the appropriate carrier atom. The methyl H atoms were refined as a rigid group, which was allowed to rotate. The structure was refined by the full-matrix least-squares method on F^2^s using the SHELXL-97 program [2]. The crystal data, together with the details concerning the data collection and structure refinement are given in Table 1 and the atomic coordinates in Table 2. The crystallographic data in the CIF form are available as electronic supplementary information from the Cambridge Crystallographic Database Centre (CCDC 1003815). Molecular illustration was prepared using ORTEP-3 for Windows [3]. Software used to prepare material for publication was WINGX [3] and PLATON [4]

Taylor et al. reported the characteristic bands in the infrared spectra of several 2-substituted 4-thiazolidinones [4]. Typically in the infra-red spectrum in the solid state shows νC = O at 1718 cm^−1^, νNH at 3015 cm^−1^ with νC = C at 1532 cm^−1^ and νC = N at 1638 cm^−1^ [43, 17, 12, 11, 16]. The 4-thiazolidinones with hydrogen attached to the nitrogen show absorption in the region 3100–3400 cm^-l^, characteristic of the N-H stretching [44]. The latter statement was based on IR data obtained for different derivatives, showing a very strong double bond C = N; absorption at 1640 cm^−1^, which is characteristic for an endocyclic C = N [45]. Furthermore, the presence/absence of an amide II band between 1500 and 1575 cm^−1^ [46] confirm the assumption that the 2-amino form h is the predominant.

## Conclusions

Thiazolidinones which belong to an important group of heterocyclic compounds have been widely explored for their applications in the field of medicine. During the last few decades this molecule has been a promising core structure for the search of new biologically active compounds due to their divers baiological potential. The aim of this study was to discuss the tautomerism of the 2-[(2,4-dimethoxyphenyl)amino]-1,3-thiazolidin-4-one. Based on the experimental X-ray analyses, FTIR spectroscopy and theoretical chemical calculations it was found that the minimum differences of energy was observed in *imino* (d)*⇋ amino* (h) pair, i.e., about 2 kcal mol^−1^. This proved that 2Z *imine* and *amine* counterpart is not experimentally accessible as isolate species. From a chemical point of view, another tautomerism such as *keto ⇋ enol* is also possible. However, among the structures deposited in the CSD database none adopts the *enol* tautomeric form. The structure activity relationship revealed that thiazolidine ring is essential for antibacterial and antiviral activity [1, 3, 39]. Our MEP analysis showed that, the compounds having E/Z configuration of *imine* has predicted higher probability of occurrence of antibacterial than its *amine* counterpart. In contrary, the *amine* spices have the higher chance to confirm antiviral activity in real pharmacological tests.

## Electronic supplementary material

Below is the link to the electronic supplementary material.

ESM 1 (DOCX 1161 kb)
