# Supplementary material for: Conformational space and vibrational spectra of 2-[(2,4-dimethoxyphenyl)amino]-1,3-thiazolidin-4-one
Source: J Mol Model. 2014 Jul 15;20(8):2366. doi: 10.1007/s00894-014-2366-6 (PMC4139587; doi:10.1007/s00894-014-2366-6)
Supplement: Supplementary file 1 — (DOCX 1161 kb) [file 894_2014_2366_MOESM1_ESM.docx]

**Title:**

**Conformational Space and Vibrational Spectra of 2-[(2,4-dimethoxyphenyl)amino]-1,3-thiazol-4(5H)-one**

**Author names and affiliations:**

Alicja Nowaczyk^a^*, Marcin Kowiel^b^, Andrzej Gzella^b^, Łukasz Fijałkowski^a^, Volodymyr Horishny^c^, Roman Lesyk^c^

^a^Department of Organic Chemistry, Faculty of Pharmacy, Collegium Medicum in Bydgoszcz, Nicolaus Copernicus University, Dr. A. Jurasza 2, 85-094 Bydgoszcz, Poland;

^b^Department of Organic Chemistry, Poznan University of Medical Sciences, ul. Grunwaldzka 6, 60-780 Poznań, Poland

^c^Department of Pharmaceutical, Organic and Bioorganic Chemistry, Faculty of Pharmacy, Danylo Halytsky Lviv National Medical University, Pekarska 69, 79010 Lviv-10, Ukraine

* **Corresponding author:**

Department of Organic Chemistry, Faculty of Pharmacy, Collegium Medicum in Bydgoszcz, Nicolaus Copernicus University, Dr. A. Jurasza 2, 85 - 094 Bydgoszcz, Poland;

Tel: +(48)(52) 5853904;

E-mail address: alicja@cm.umk.pl

Table S1. *Refcodes and molecular structures of 2-amino-1,3-thiazolidin-4-ones found in CSD Cambridge, Version 5.35 [1]*

| No. | Refcodes | No. | Refcodes | No. | Refcodes | No. | Refcodes |
| --- | --- | --- | --- | --- | --- | --- | --- |
| 1. | EKELEL | 7. | INMTZO | 12. | PTHAZO10 | 17. | ULACAM |
|  |  |  |  |  |  |  |  |
| 2. | FIVPIJ | 8. | JOBGOW | 13. | SALYOT | 18. | VELBEU |
|  |  |  |  |  |  |  |  |
| 3. | FOWQOY | 9. | KUKZUM | 14. | SINQOW | 19. | VEQFAA |
|  |  |  |  |  |  |  |  |
| 4. | IHUFAS | 10. | PACPIU | 15. | SINQUC | 20. | WOSMAS |
|  |  |  |  |  |  |  |  |
| 5. | IMPTHA12 | 11. | PATAZO | 16. | TEBDAH | 21. | YUQCAP |
|  |  |  |  |  |  |  |  |
| 6. | IMTAZO01 |  |  |  |  |  |  |
|  |  |  |  |  |  |  |  |

Table S2. *Refcodes and molecular structures of 2-imino-1,3-thiazolidin-4-ones found in CSD Cambridge, Version 5.35 [1]*

| No. | Refcodes | No. | Refcodes | No. | Refcodes | No. | Refcodes |
| --- | --- | --- | --- | --- | --- | --- | --- |
| 1. | EHITZO | 4. | HEGMIR | 6. | ROMXUN | 8. | ULACEQ |
|  |  |  |  |  |  |  |  |
| 2. | HEGMAJ | 5. | HEGMOX | 7. | SOHHIH | 9. | VAMPUW |
|  |  |  |  |  |  |  |  |
| 3. | HEGMEN |  |  |  |  |  |  |
|  |  |  |  |  |  |  |  |

Table S3. *Refcodes and molecular structures of N3-substituted 2-imino-1,3-thiazolidin-4-one derivatives found in CSD Cambridge, Version 5.35 [1]*

| No. | Refcodes | No. | Refcodes | No. | Refcodes | No. | Refcodes |
| --- | --- | --- | --- | --- | --- | --- | --- |
| 1. | ADUDIO | 14. | IMMAZO10 | 26. | PAWNOS | 38. | XAYYON |
|  | 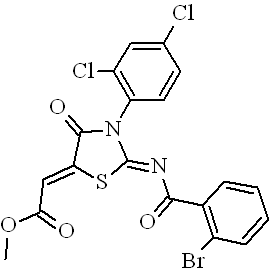 |  | 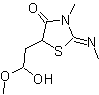 |  | 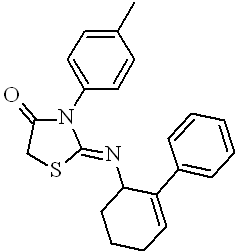 |  | 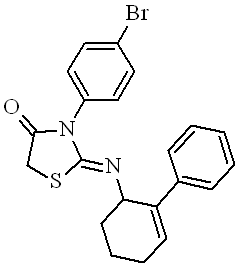 |
| 2. | AZOPIP | 15. | ITILIG | 27. | PECRIA | 39. | XAYZII |
|  | 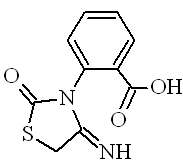 |  | 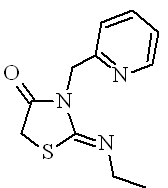 |  | 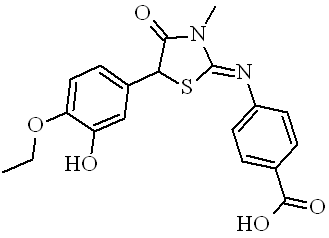 |  | 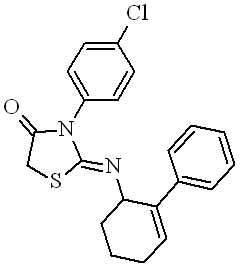 |
| 3. | BIVWUZ | 16. | KETRIL | 28. | PIMPTZ10 | 40. | XITFIP |
|  | 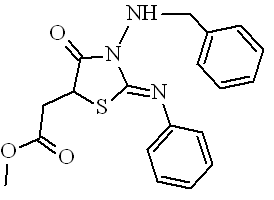 |  | 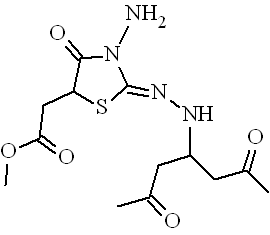 |  | 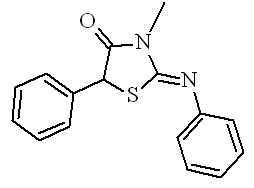 |  | 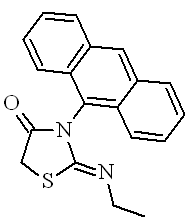 |
| 4. | CAPGIK | 17. | KISBOE | 29. | QONWAS | 41. | XUKGEQ |
|  | 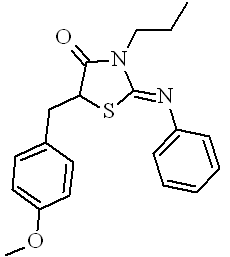 |  | 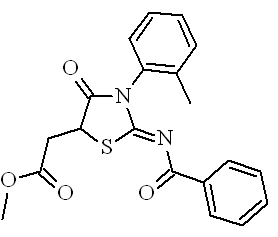 |  | 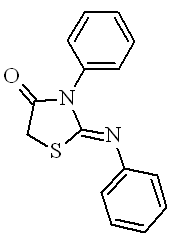 |  | 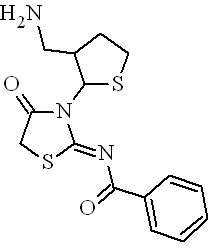 |
| **5.** | COTPUX | 18. | LEFFEJ | 30. | QONWEW | 42. | XUKGIU |
|  | 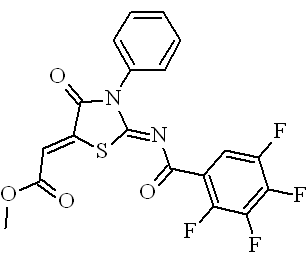 |  | 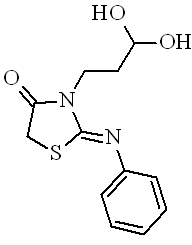 |  | 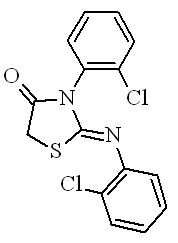 |  | 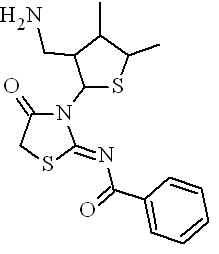 |
| 6. | CUJVAE | 19 | LEGNIW | 31 | RAJJAP | 43 | XUKGOA |
|  | 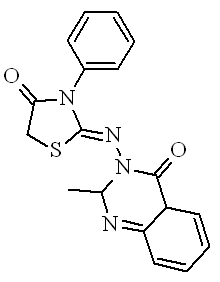 |  | 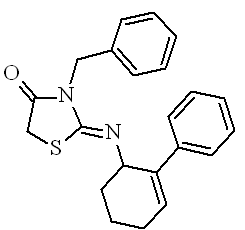 |  | 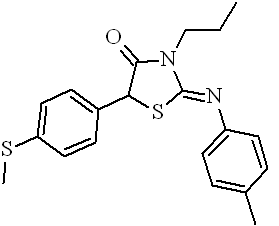 |  | 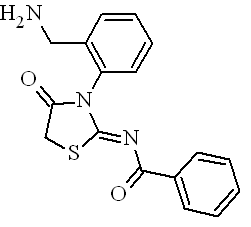 |
| 7. | DIGTOD | 20 | MOSTEU | 32 | SANXUB | 44 | YAXLEQ |
|  | 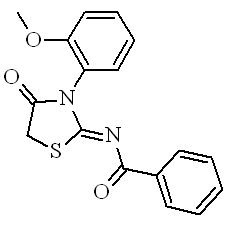 |  | 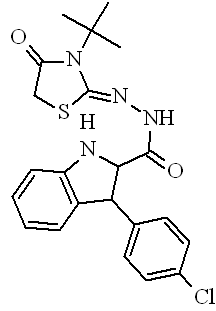 |  | 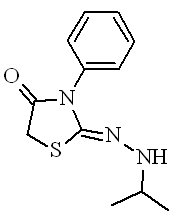 |  | 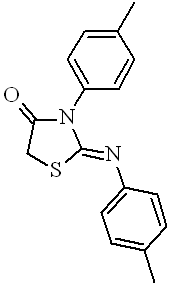 |
| 8. | DIYQIM | 21. | OGEZUV | 33. | SIXFOV | 45. | YEKGIG |
|  | 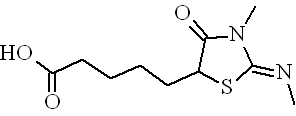 |  | 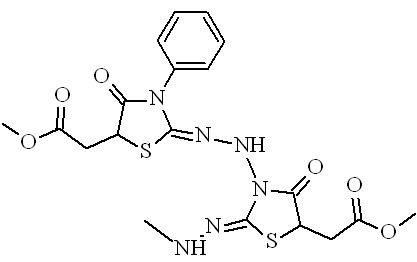 |  | 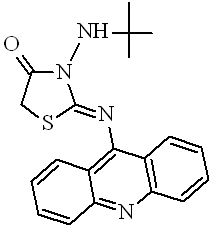 |  | 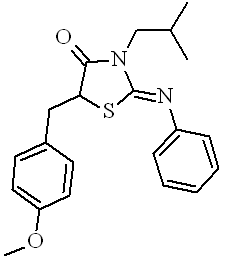 |
| 9. | EVEQOM | 22. | OGIBAH | 34. | SOHHON | 46. | YIFMAC |
|  | 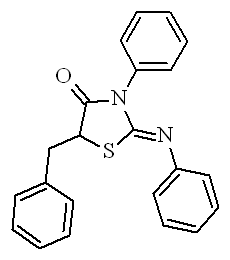 |  | 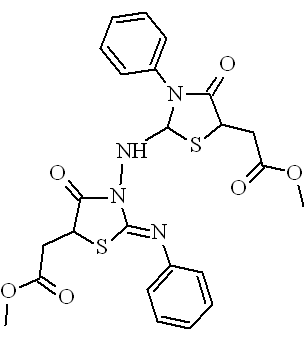 |  | 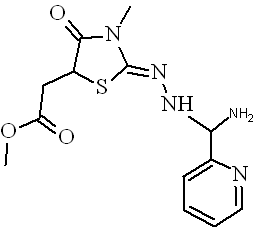 |  | 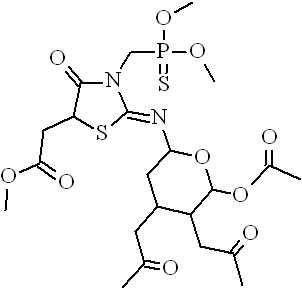 |
| 10. | FIHSAR | 23. | OYEXUM | 35. | VELBIY | 47. | YOYREJ |
|  | 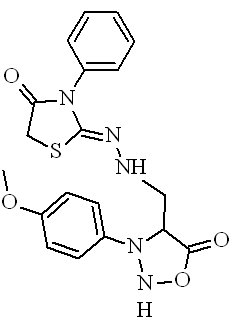 |  | 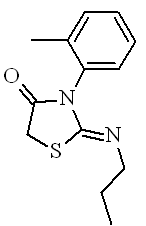 |  | 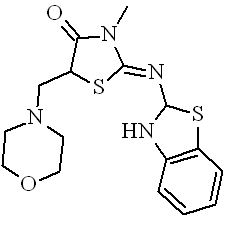 |  | 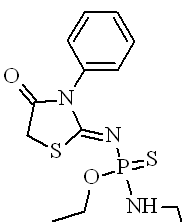 |
| 11. | FOBQAP | 24. | OYEYAT | 36. | WADZOS | 48. | YUQDOE |
|  | 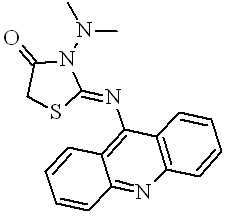 |  | 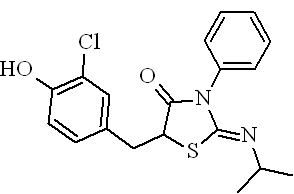 |  | 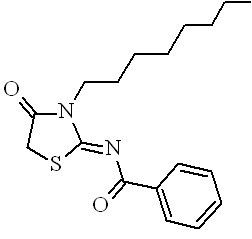 |  | 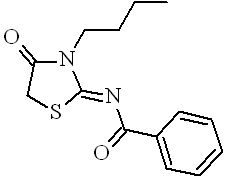 |
| 12. | HEGMUD | 25. | OYEYEX | 37. | XAYYEC | 49. | ZEWCAH |
|  | 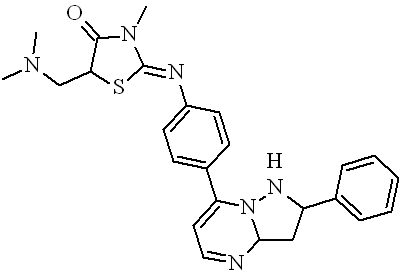 |  | 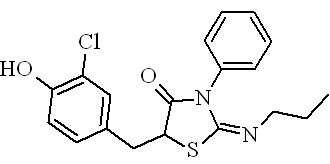 |  | 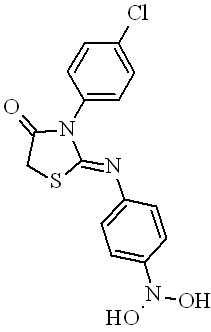 |  | 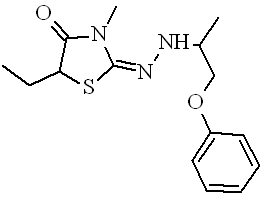 |
| 13. | HEGNAK |  |  |  |  |  |  |
|  | 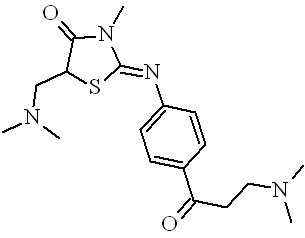 |  |  |  |  |  |  |

Table S4. *Refcodes and molecular structures of 2-amino-1,3-thiazolidin-4-one derivatives with (N6) tertiary amino group found in CSD Cambridge, Version 5.35 [1]*

| No. | Refcodes | No. | Refcodes | No. | Refcodes | No. | Refcodes |
| --- | --- | --- | --- | --- | --- | --- | --- |
| 1. | AWUPEO | 6. | HIDJIO | 11. | LEQZIS | 15. | REZCIJ |
|  | 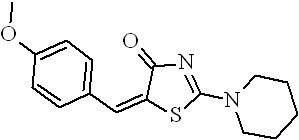 |  | 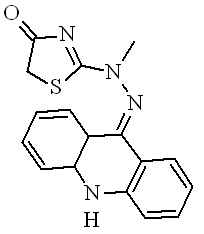 |  | 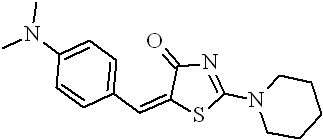 |  | 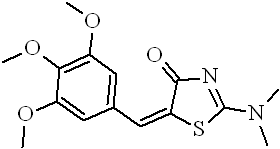 |
| 2. | BAHGOI | 7. | IZERIP | 12. | LOBGIS | 16. | UYIPIC |
|  | 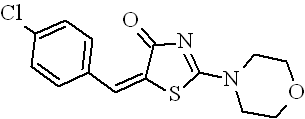 |  | 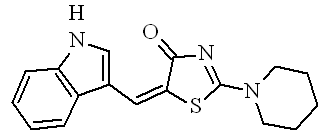 |  | 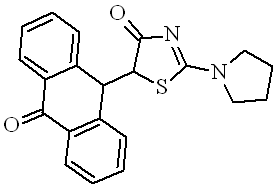 |  | 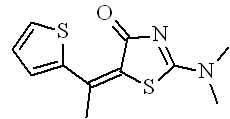 |
| 3. | CELLAI | 8. | IZEROV | 13. | MCMPTZ | 17. | YAGFET |
|  | 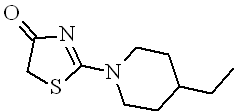 |  | 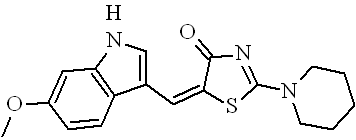 |  | 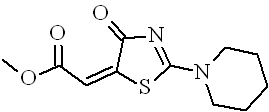 |  | 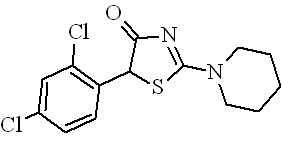 |
| 4. | ERATUM | 9. | KUQKUD | 14. | PMPTZO | 18. | YEJTAK |
|  | 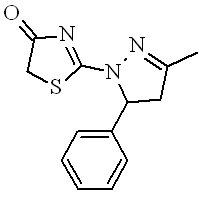 |  | 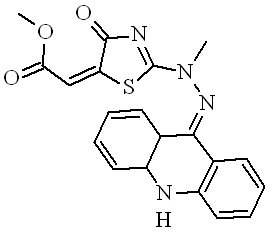 |  | 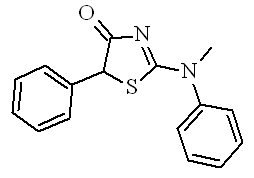 |  | 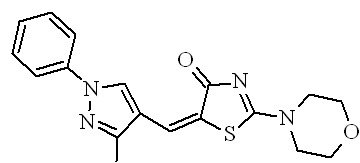 |
| 5. | GACXUF | 10. | LENTEF |  |  |  |  |
|  | 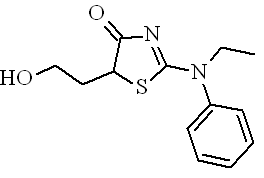 |  | 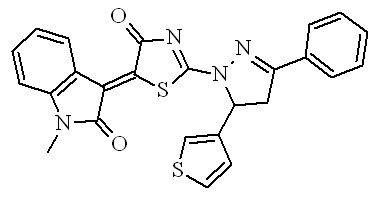 |  |  |  |  |

Table S5. *Statistics for bond lengths C2-N3 and C2-N6 of 2-amino(imino)-1,3-thiazolidin-4-one derivatives (CSD Cambridge, Version 5.35, R < 7.00%)*

| 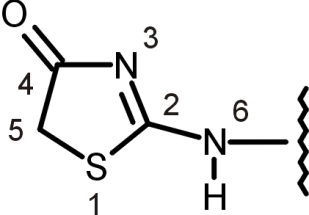 | | | | 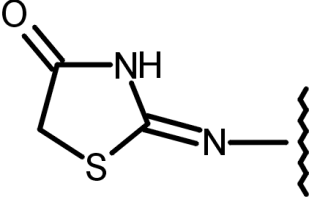 | | | |
| --- | --- | --- | --- | --- | --- | --- | --- |
| Refcodes [30] | Bond lengths (Å) | | | Refcodes [30] | Bond lengths (Å) | | |
|  | C2−N3 | C2−N6 | *R* (%) |  | C2−N3 | C2−N6 | *R* (%) |
| EKELEL | 1,327 | 1,313 | 2.93 | EHITZO | 1.387 | 1.282 | 4.40 |
| FIVPIJ | 1,318 | 1,318 | 2.92 | HEGMAJ | 1.372 | 1.268 | 6.83 |
| FOBQOY | 1,328 | 1,317 | 2.90 |  | 1.362 | 1.281 |  |
| IHUFAS | 1,320 | 1,310 | 4.70 | HEGMEN | 1.381 | 1.282 | 3.05 |
| IMPTHA12 | 1,341 | 1,316 | 6.50 | HEGMIR | 1.363 | 1.296 | 3.17 |
|  | 1,336 | 1,322 |  | HEGMOX | 1.369 | 1.277 | 4.70 |
| IMTAZO01 | 1,333 | 1,317 | 2.09 |  | 1.371 | 1.284 |  |
| INMTZO | 1,337 | 1,294 | 4.70 | ROMXUN | 1.367 | 1.278 | 6.24 |
| JOBGOW | 1,330 | 1,298 | 4.50 | ULACEQ | 1.390 | 1.260 | 4.81 |
| KUKZUM | 1,327 | 1,317 | 2.79 | VAMPUV | 1.380 | 1.292 | 5.84 |
|  | 1,317 | 1,320 |  |  |  |  | |
| PACPIU | 1,331 | 1,318 | 3.48 |  |  |  | |
|  | 1,326 | 1,321 |  |  |  |  | |
| PTHAZO10 | 1,315 | 1,344 | 4.00 |  |  |  | |
|  | 1,322 | 1,334 |  |  |  |  | |
| SALYOT | 1,326 | 1,315 | 3.11 |  |  |  | |
| SINQOW | 1,319 | 1,309 | 4.35 |  |  |  | |
|  | 1,319 | 1,311 |  |  |  |  | |
| SINQUC | 1,322 | 1,309 | 3.98 |  |  |  | |
|  | 1,316 | 1,316 |  |  |  |  | |
| TEBDAH | 1,320 | 1,321 | 5.19 |  |  |  | |
| ULACAM | 1,312 | 1,295 | 4.88 |  |  |  | |
| VELBEU | 1,322 | 1,339 | 3.50 |  |  |  | |
| VEQFAA | 1,328 | 1,299 | 5.65 |  |  |  | |
| WOSMAS | 1,329 | 1,307 | 3.50 |  |  |  | |
| YUQCAP | 1,324 | 1,310 | 4.30 |  |  |  | |
| **Mean value*** | **1.325(1)** | **1.315(2)** | | **Mean value*** | **1.374(3)** | **1.280(2)** | |
| **Sample standard deviation** | **0.007** | **0.012** | | **Sample standard deviation** | **0.009** | **0.010** | |

** Number in brackets is standard error of mean in the unit of the least significant digit of the preceding numer.*

*Structures not included in the statistics for bond lengths C2-N3 and C2-N6 with their R values:* IMPTHA01, 11.00; PATAZO, 12.00; GACXOZ, 5.89; HEGLUC, 6.23; IMTAZO, 11.50; SOHHIH, 9.82. *The X–ray studies [5] showed that the structures denoted with refcodes GACXOZ, HEGLUC exist in the crystal as the tautomer with carbonyl-imine moiety in the five-membered heterocyclic ring and an exocyclic amine N atom rather than the previously reported tautomer with the secondary amide group and an exocyclic imine N atom [6,7].*

Table S6. *Statistics for bond lengths C2-N3 of N3-substituted 2-imino-1,3-thiazolidin-4-ones [1]*

| 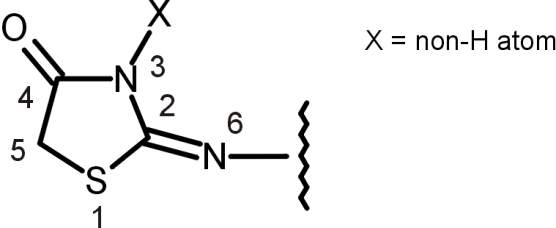 | | | | | |
| --- | --- | --- | --- | --- | --- |
| No. | Refcode | Bond length (Å) C2−N3 | No. | Refcode | Bond length (Å) C2−N3 |
| 1. | ADUDIO | 1.390 | 34. | HEGMAJ | 1.372 |
| 2. | ANIWAW | 1.374 |  |  | 1.362 |
| 3. | AVUMUZ | 1.396 | 35. | HEGMEN | 1.381 |
| 4. | AVUNEK | 1.372 | 36. | HEGMIR | 1.363 |
| 5. | AWUPAK | 1.372 | 37. | HEGMOX | 1.371 |
|  |  | 1.371 |  |  | 1.369 |
| 6. | AZOPIP | 1.406 | 38. | HEGMUD | 1.377 |
| 7. | BIVWUZ | 1.400 | 39. | HEGNAK | 1.369 |
| 8. | BOQKIB | 1.345 | 40. | HOLHIA | 1.380 |
| 9. | CAPGIK | 1.377 | 41. | HOTSAL | 1.375 |
|  |  | 1.374 | 42. | IDUWUA | 1.395 |
| 10. | CESCAF | 1.365 | 43. | IKICUA | 1.384 |
| 11. | COTPUX | 1.373 | 44. | ILIQAV | 1.380 |
|  |  | 1.381 | 45. | IMMAZO10 | 1.383 |
|  |  | 1.360 |  |  | 1.396 |
|  |  | 1.380 | 46. | IYAYOW | 1.395 |
| 12. | CUHHIX | 1.364 | 47. | ITILIG | 1.409 |
| 13. | DIGTOD | 1.377 | 48. | KADXOC | 1.374 |
| 14. | DIWSIM | 1.369 | 49. | KETRIL | 1.372 |
| 15. | DIYQIM | 1.396 | 50. | KIMRUV | 1.357 |
| 16. | EHITZO | 1.387 | 51. | KUSQUL | 1.363 |
| 17. | EKEZID | 1.383 | 52. | KUTHAJ | 1.384 |
| 18. | ELEYEZ | 1.382 | 53. | LEFFEJ | 1.400 |
| 19. | ELEZAW | 1.375 | 54. | LEGNIW | 1.402 |
| 20. | ELEZEA | 1.391 | 55. | MAPBIP | 1.384 |
| 21. | ETIHAQ | 1.377 | 56. | MAVKIE | 1.392 |
| 22. | EVEQOM | 1.394 | 57. | METWOY | 1.385 |
|  |  | 1.396 | 58. | METWUE | 1.377 |
| 23. | FIHSAR | 1.411 | 59. | MOSTEU | 1.393 |
| 24. | FOBQAP | 1.397 | 60. | OCECIK | 1.375 |
| 25. | FONMIF | 1.385 | 61. | OGIBAH | 1.386 |
| 26. | FUMDOH | 1.380 |  |  | 1.388 |
| 27. | FUMDUN | 1.391 |  |  | 1.404 |
| 28. | GAGFAX | 1.375 |  |  | 1.382 |
|  |  | 1.356 | 62. | OJIDOA | 1.379 |
| 29. | GAMGUX | 1.394 | 63. | OMIXOX | 1.355 |
| 30. | GAQPAR | 1.374 | 64. | OMIYOY | 1.355 |
| 31. | GAVLOF | 1.377 | 65. | OYEXOG | 1.381 |
| 32. | HAKPEQ | 1.388 |  |  | 1.389 |
| 33. | HARTIF | 1.382 |  |  |  |

Table 6. *continued*

| No. | Refcode | Bond length (Å) C2−N3 | No. | Refcode | Bond length (Å) C2−N3 |
| --- | --- | --- | --- | --- | --- |
| 66. | OYEXUM | 1.412 | 92. | VAMPUW | 1.380 |
|  |  | 1.406 | 93. | VATLOS | 1.371 |
| 67. | OYEYAT | 1.405 |  |  | 1.387 |
| 68. | OYEYEX | 1.408 | 94. | VATMAF | 1.377 |
| 69. | PAWNOS | 1.407 | 95. | WADZOS | 1.383 |
| 70. | PECRIA | 1.387 | 96. | WEVZEC | 1.376 |
| 71. | POHKUT | 1.376 | 97. | XAHKUO | 1.383 |
| 72. | PONSER | 1.375 | 98. | XAHLAV | 1.381 |
| 73. | PONWUL | 1.386 | 99. | XAYYEC | 1.404 |
| 74. | PUJRIW | 1.366 | 100. | XAYYON | 1.409 |
| 75. | QECZAB | 1.376 | 101. | XAYZII | 1.410 |
| 76. | QONWAS | 1.403 | 102. | XETKOX | 1.377 |
| 77. | QONWEW | 1.428 |  |  | 1.374 |
| 78. | REQNIL | 1.385 | 103. | XETKOX01 | 1.376 |
| 79. | ROMXUN | 1.367 | 104. | XITFIP | 1.415 |
| 80. | SAHKIX | 1.371 | 105. | XUKGEQ | 1.398 |
| 81. | SANXUB | 1.388 | 106. | XUKGIU | 1.394 |
| 82. | SEDJIW | 1.364 | 107. | XUKGOA | 1.381 |
| 83. | SEDLAQ | 1.346 | 108. | YAJGIB | 1.369 |
| 84. | SESHII | 1.380 | 109. | YAXLEQ | 1.404 |
| 85. | SESHUU | 1.379 | 110. | YAXYED | 1.370 |
| 86. | SESJAC | 1.379 | 111. | YAYHAJ | 1.355 |
| 87. | SEZDEH | 1.369 | 112. | YEKGIG | 1.378 |
| 88. | SISMUD | 1.386 | 113. | YIFMAC | 1.397 |
|  |  | 1.383 | 114. | YIJFED | 1.346 |
| 89. | SIXFOV | 1.399 | 115. | YOYREJ | 1.390 |
|  |  | 1.400 | 116. | YUQDOE | 1.372 |
| 90. | SOHHON | 1.391 | 117. | ZAPSUG | 1.390 |
| 91. | ULACEQ | 1.390 |  |  |  |
| **Mean value*** | | | **1.383(1)** | | |
| **Sample standard deviation** | | | **0.015** | | |

* *Number in brackets is standard error of mean in the unit of the least significant digit of the preceding numer.*

Table S7. *Statistics for torsion angles S1-C2-N6-X of 2-imino-1,3-thiazolidin-4-one derivatives
(CSD Cambridge, Version 5.35, R < 7.00%)*

| 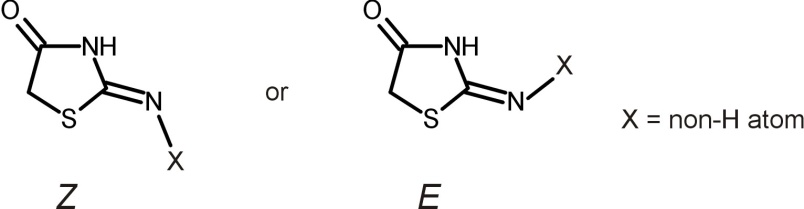 | |
| --- | --- |
| Refcodes [30] | S1−C2−N6−X |
| EHITZO | -0.12 |
| HEGMAJ | -1.48 |
|  | -6.53 |
| HEGMEN | -2.06 |
| HEGMIR | 1.18 |
| HEGMOX | 1.99 |
|  | 2.27 |
| ROMXUN | 1.14 |
| ULACEQ | -1.80 |
| VAMPUV | -1.14 |

Table S8. *The atomic charge for tautomers* ***c, d, h*** *respectively.*

| *(2E) imine* | 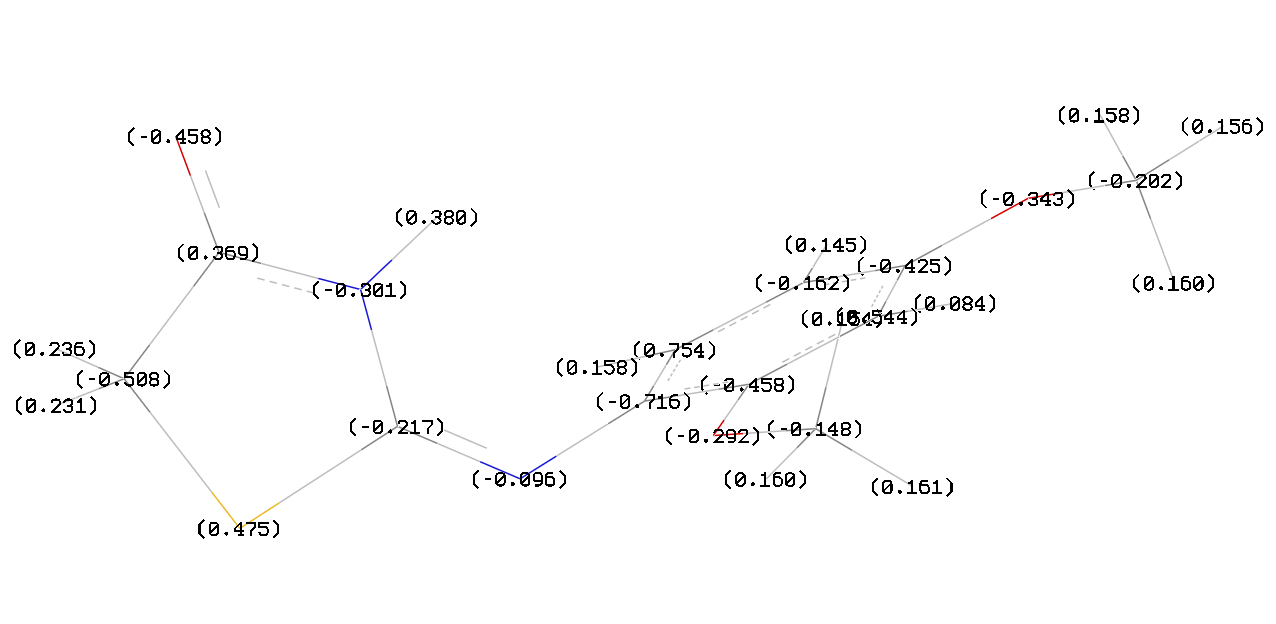 |
| --- | --- |
| (2Z) *imine* | 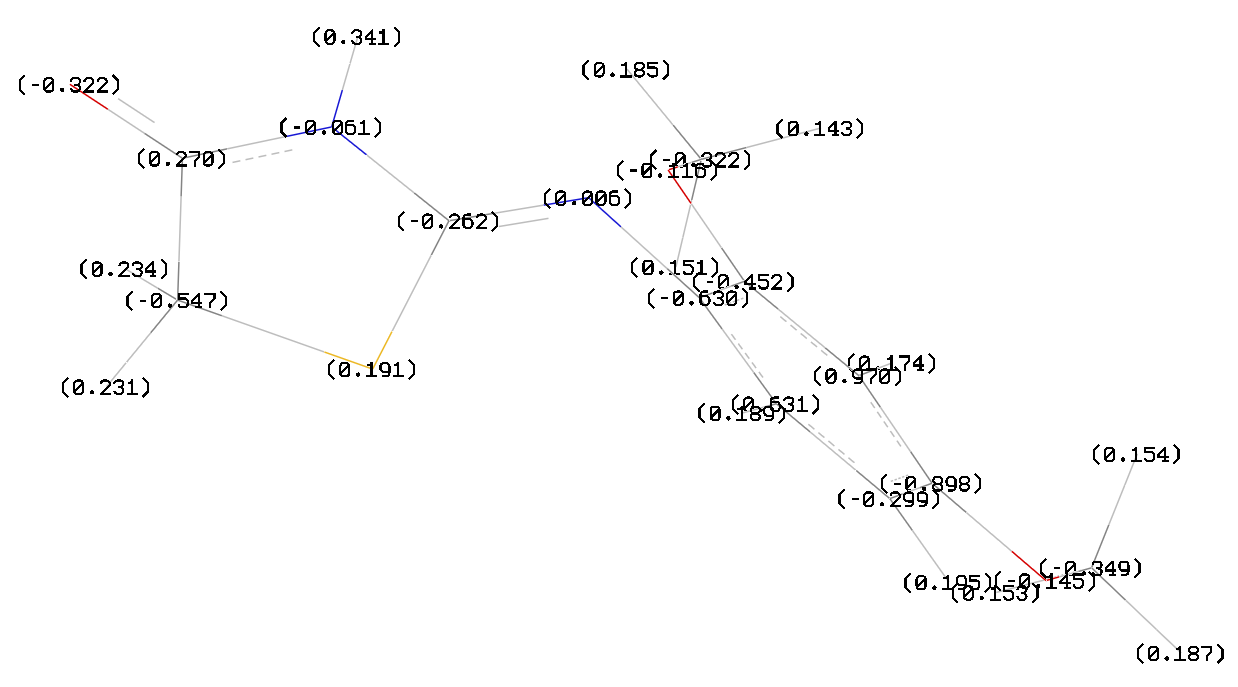 |
| *2-amine* | 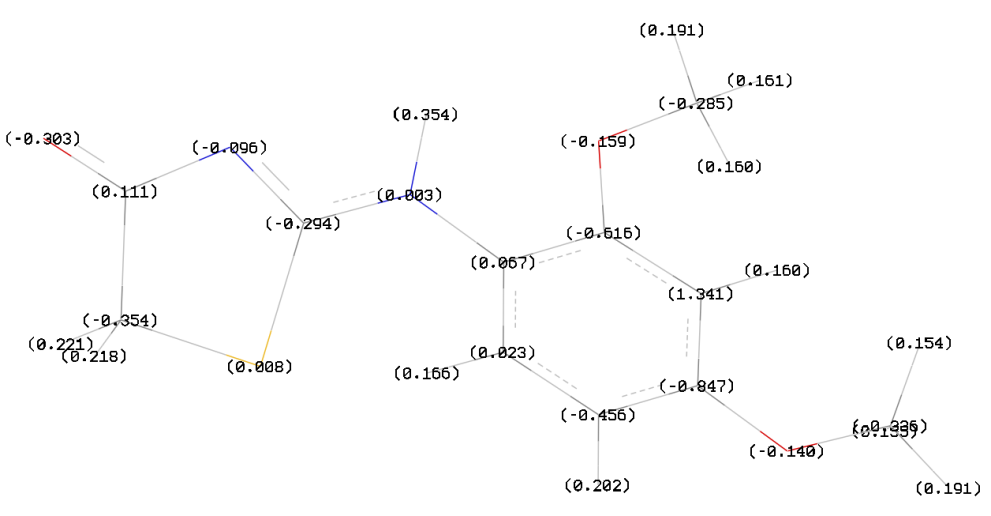 |

*X-ray crystallography*

X-ray diffraction measurements were carried out on an Agilent Xcalibur A diffractometer [1]. The structure of **1** was solved by direct methods using the SHELXS-97 program [2]. Except for the amine H atom, which was refined freely the remaining H atoms were positioned geometrically and were refined within the riding model approximation, with C–H = 0.96 Å (CH_3_), 0.97 Å (CH_2_), 0.93 Å (C*_ar_*H) and *U*_iso_ (H) values were constrained to be 1.2 (1.5 for methyl group) times *U*_eq_ of the appropriate carrier atom. The methyl H atoms were refined as a rigid group, which was allowed to rotate. The structure was refined by the full-matrix least-squares method on F^2^s using the SHELXL-97 program [2] . The crystal data, together with the details concerning the data collection and structure refinement are given in Table 1 and the atomic coordinates in Table 2. The crystallographic data in the CIF form are available as Electronic Supplementary Information from the Cambridge Crystallographic Database Centre (CCDC 1003815). Molecular illustration was prepared using ORTEP-3 for Windows [3]. Software used to prepare material for publication was WINGX [3] and PLATON [4].

Table 2. Fractional atomic coordinates and U_eq_ with esds (in parenthesis) for **1**

Atom *x*/*a* *y*/*b z*/*c* *U*_eq_

S1 0.660734(15) 0.22164(2) 0.21553(3) 0.01584(7)

C2 0.59322(6) 0.35562(9) 0.12370(11) 0.01267(15)

N3 0.50198(5) 0.33392(8) 0.07046(10) 0.01477(15)

C4 0.47706(6) 0.20298(9) 0.09243(11) 0.01518(16)

C5 0.55909(6) 0.11438(9) 0.16734(12) 0.01718(17)

N6 0.63270(5) 0.47397(8) 0.10697(10) 0.01398(14)

C7 0.73068(6) 0.50331(9) 0.15356(11) 0.01301(15)

C8 0.78267(6) 0.55422(9) 0.03102(11) 0.01310(15)

C9 0.87761(6) 0.59364(9) 0.07162(11) 0.01507(16)

C10 0.92013(6) 0.57732(9) 0.23423(12) 0.01576(16)

C11 0.86950(6) 0.52375(10) 0.35594(11) 0.01609(17)

C12 0.77429(6) 0.48919(9) 0.31511(11) 0.01514(16)

O13 0.73442(5) 0.56009(7) - 0.12362(8) 0.01722(14)

C14 0.77689(7) 0.63452(12) -0.24591(12) 0.0225(2)

O15 1.01253(5) 0.61259(9) 0.28638(10) 0.02465(17)

C16 1.05974(7) 0.69796(12) 0.18180(16) 0.0271(2)

O17 0.39728(5) 0.15797(8) 0.05355(10) 0.02246(15)

**REFERENCES**

1. Agilent (2011). CrysAlis PRO. Oxford Diffraction Ltd, Yarnton, England.
2. Sheldrick, G. M. (2008). Acta Cryst. A64, 112–122.
3. Farrugia, L. J. (2012). J. Appl. Cryst. 45, 849–854.
4. Spek, A. L. (2009). Acta Cryst. D65, 148–155.
5. Gzella A. K., Kowiel M., Suseł A., Wojtyra M. N., Lesyk R., *Acta Cryst.*, 2014; Section C, submitted for publication; reference code WQ3065.
6. Vana J., Hanusek J., Ruzicka A., Sedlak M., *J. Heterocycl. Chem.,* 2009; **46,** 635-639; refcode: GACXOZ.
7. Behbehani H., Mohamed Ibrahim H *Molecules,* 2012; **17**, 6362-6385; refcodes: HEGLUC, HEGMAJ, HEGMEN, HEGMIR, HEGMOX.
